# Extracellular vesicles-mediated noncoding RNAs transfer in cancer

**DOI:** 10.1186/s13045-017-0426-y

**Published:** 2017-02-23

**Authors:** Pei Ma, Yutian Pan, Wei Li, Chongqi Sun, Jie Liu, Tongpeng Xu, Yongqian Shu

**Affiliations:** 1 Department of Oncology, the First Affiliated Hospital of Nanjing Medical University, 300 Guangzhou Road, Nanjing, 210029 People’s Republic of China; 2 Jiangsu Key Lab of Cancer Biomarkers, Prevention and Treatment, Collaborative Innovation Center for Cancer Personalized Medicine, Nanjing Medical University, Nanjing, People’s Republic of China

**Keywords:** Extracellular vesicles, Noncoding RNAs, Cancer, Mechanism

## Abstract

Extracellular vesicles (EVs) are small membranous vesicles secreted from numerous cell types and have been found involved in cell-to-cell communication by transferring noncoding RNAs (ncRNAs) including microRNAs, long noncoding RNAs, and circular RNAs. Emerging evidence shows that EV-associated ncRNAs play important roles in a wide range of diseases, particularly in cancer where they function through regulating protein expression of the pivotal genes that make contributions to tumorigenesis. Given their stability and abundance in serum, EV-associated ncRNAs can act as new diagnostic biomarkers and new therapeutic targets for cancer. Herein, we review the properties of EV-associated ncRNAs, their functions, and potential significance in cancer.

## Background

Extracellular vesicles (EVs) are small lipid bilayered vesicles released by a wide range of normal or diseased cells. Three main types of EVs are exosomes, microvesicles, and apoptotic bodies which are distinguished on the basis of their size and biogenesis [1, 2]. Exosomes are 40 to 100 nm in diameter and are released by multivesicular bodies. Microvesicles are 50 to 1000 nm in diameter and are formed by budding directly from the plasma membrane. Apoptotic bodies are 800 to 5000 nm in diameter and are derived by apoptotic cells [3–5]. The current golden standard method for separating and purifying EVs, differential ultracentrifugation, is incapable of distinguishing between exosomes and microvesicles [6]. In order to avoid unnecessary confusion, we used the term “EVs” in this review.

Noncoding RNAs (ncRNAs) refer to RNAs that cannot be translated into proteins. MicroRNAs (miRNAs) are the most widely studied class of ncRNAs with length of ~22 nucleotides, which mediate post-transcriptional gene silencing by controlling the translation of mRNA into proteins in animals [7, 8]. Long noncoding RNAs (lncRNAs) are a heterogeneous group of noncoding transcripts that make up the largest portion of the mammalian noncoding transcriptome with a length of more than 200 nucleotides [9]. LncRNAs are known to regulate gene expression via various mechanisms. For example, they can mediate epigenetic modifications of DNA by recruiting chromatin-remodeling complexes to specific loci [10, 11]. Other types of ncRNAs include ultraconserved regions (T-UCRs), small nucleolar RNAs (snoRNAs), PIWI-interacting RNAs (piRNAs) as well as circular RNAs (circRNAs), which might also contribute to the development of many different human disorders [9, 12]. Currently, ncRNAs are found to have diverse biological regulatory functions and dysregulated expression of ncRNAs is closely associated with development of diseases including cancer [12].

EVs that were initially considered as garbage bags for abandoned membrane parcels and molecular fragments were first recognized as being closely related to the function of the immune system with the finding of the role of exosomes in the presentation of B lymphocyte antigens in 1996 by Raposo et al. [13]. In the 2010s, researchers found that miRNA and mRNA can be loaded as “goods” in EVs [14]. In recent years, EVs are discovered to serve as “communication shuttles” between cells and transduct signals between cells [15].

**Fig. 1:**
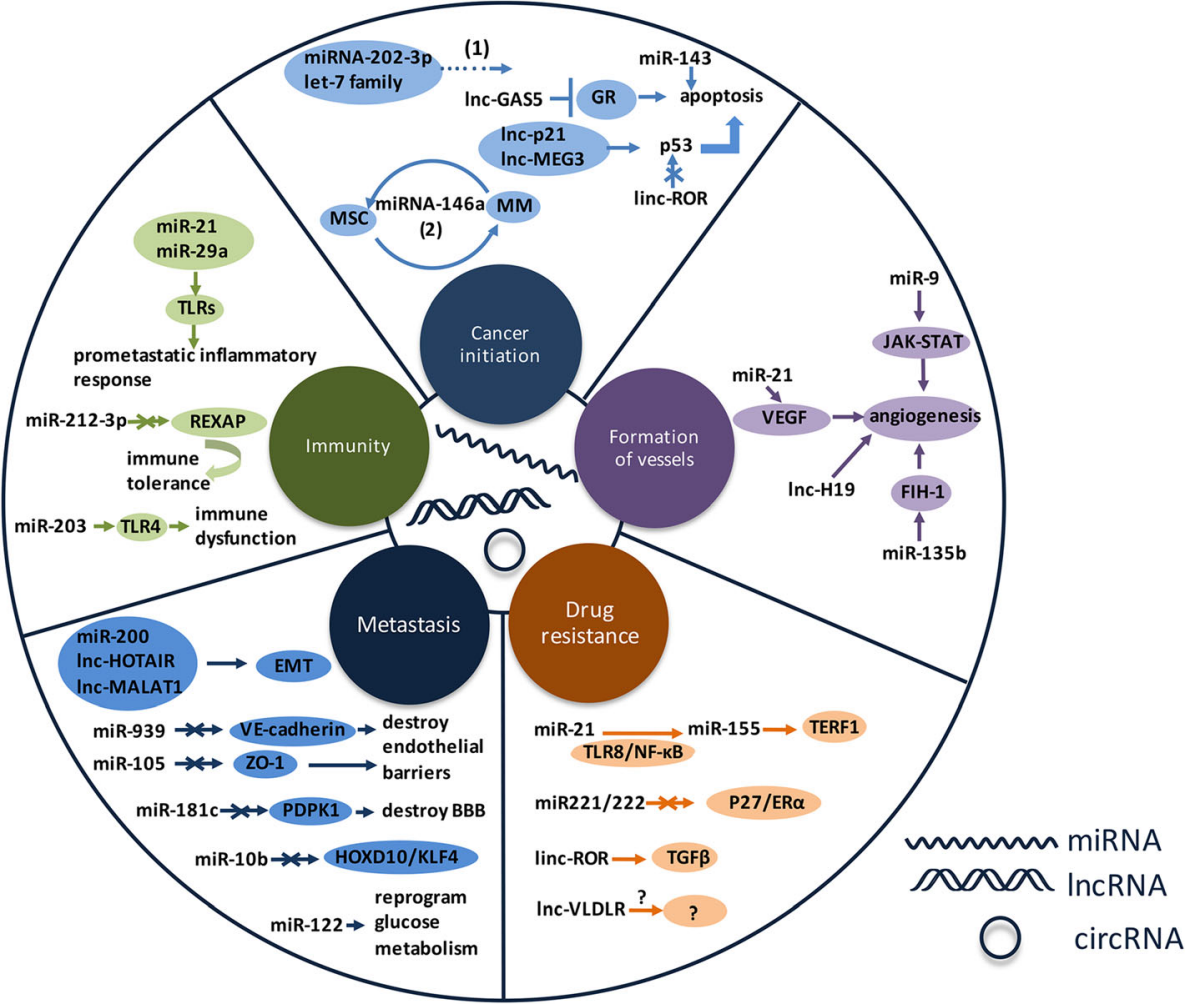
EV-associated ncRNAs in cancer. EV-associated ncRNAs contribute to the five types of function in cancer. Selected examples of EV-associated ncRNAs and their mechanisms are shown in cancer initiation, formation of vessels, drug resisitance, metastasis, and immunity. (*1*) Cancerous cells might discard anti-tumorigenic miRNAs via EVs to stimulate cancer initiation and progression. (*2*) There exists a positive feedback loop between MM cells and MSC that MM cells promote the increase of miR146a in MSC leading to more cytokine secretion, which in turn favors MM cell growth and migration

Herein, we will discuss how ncRNAs derived from EVs participate in tumorigenesis, invasion, metastasis, and drug resistance and how to use them as new diagnostic biomarkers and therapeutic targets (Fig. 1).

### EV-associated miRNAs in cancer

MiRNAs are a class of small noncoding RNA molecules that can regulate many genes by binding to noncoding regions of target mRNAs, post-transcriptionally lowering mRNAs and proteins [7]. Secreted miRNAs were first identified in human serum and have also been found in several biological fluids including saliva, breast milk, and urine [16]. Recently, as shown by Montecalvo and colleagues, miRNAs transferred by EVs can repress mRNAs in target cells, indicating their role as cell-to-cell communication shuttles and thus having an influence on tumorigenesis and tumor development through various mechanisms [16]. Therefore, EV-associated microRNAs can both promote and suppress tumorigenesis and development, which depends on the function of their target mRNAs and protein product (Table 1) [17].

**Table 1:** EV-associated miRNAs in cancer

miRNA	Cancer type	Biological function	Mechanism	Refs
miR-202-3p	CLL	Suppress cancer initiation	Tumors discard these miRNAs via EVs to	[27]
let-7 family	Gastric cancer	Suppress cancer initiation	Promote cancer initiation	[21]
miR-146a	MM	Favor MM cell growth	Elevate several cytokines and chemokines	[25]
miR-21	Lung cancer	Promote angiogenesis	Elevate levels of VEGF	[30]
miR-21	Lung cancer	Regulate immunity	Bind as ligands to TLRs in immune cells	[53]
miR-21	Ovarian cancer	Suppress apoptosis	Bind to its target APAF1	[63]
miR-9	Breast cancer	Promote angiogenesis	Activate JAK-STAT pathway	[36]
miR-135b	MM	Promote angiogenesis	Target HIF-1	[37]
miR-939	Breast cancer	Destroy endothelial barriers	Downregulate VE-cadherin	[38]
miR-105	Breast cancer	Destroy endothelial barriers	Target the tight junction protein ZO-1	[39]
miR-10b	Breast cancer	Promote cell invasion	Suppress its target genes HOXD10 and KLF4	[40]
miR-181c	Breast cancer	Destroy BBB	Downregulate its target gene PDPK1	[43]
miR-200	Breast cancer	Promote metastasis	Regulate MET process	[47]
miR-122	Breast cancer	Promote metastasis	Reprogram glucose metabolism	[49]
miR-29a	Lung cancer	Regulate immunity	Bind as ligands to TLRs in immune cells	[53]
miR-203	PC	Cause immune dysfunction	Regulate TLR4	[54]
miR-212-3p	PC	Induce immune tolerance	Downregulate REXAP expression	[56]
miR-221/222	Breast cancer	Enhance drug resistance	Reduce target gene expression of P27 and ERa	[56]
miR-21/155	Neuroblastoma	Enhance drug resistance	Function as exosomic miR-21/ TLR8/NF-кB/exosomic miR-155/TERF1 axis	[57]
miR-143	Prostate cancer	Inhibit cell growth	Act as a death signal in cell-competitive process	[62]

Clinical features including predict treatment-free survival (TFS) and overall survival (OS) are correlated to the function of EV-associated miRNAs in cancer. For example, miR-150 can be released into the extracellular space via EVs and it has been shown that among chronic lymphocytic leukemia (CLL) patients, a low cellular miR-150 expression level is associated with tumor burden, disease aggressiveness, and poor prognostic factors while a high level of serum miR-150 is associated with tumor burden markers and some markers of poor prognosis in contrast. Similarly, cellular and serum miR-150 can also influence TFS and OS in an opposite manner: patients with low cellular/serum miR-150 levels have median TFS of 40/111 months compared with high-level patients who have a median TFS of 122/60 months (*P* < 0.0001/*P* = 0.0066). Similar results have been observed for OS [18].

### miRNAs transferred by tumor cell-derived EVs in cancer

Evidence has begun to accumulate that tumor cells have the ability of constitutively secreting a variety of EVs especially containing miRNAs, which has been found to potentially exert a paracrine influence on the surrounding cells to promote proliferation, induce angiogenesis, affect tumor immunity, and result in drug resistance. They can also act on distant organs in an endocrine fashion, which could have profound effect on metastasis [19, 20].

The first step to study EV-associated miRNAs is isolating EVs from cultured cell lines and validating their quality by analyses of transmission electron microscopy and western blotting. Secondly, RNAs are isolated from cells and culture media, and profiles of miRNA fractions are obtained using microarray analysis. Thirdly, we can observe the abundance of miRNAs in both the intracellular and extracellular fractions by comparing signal intensities of microarray data and the following validation using RT-PCR analysis [21]. We can also study the changes of biological or pathological properties in their target cells and the underlying mechanisms through which they influence cancer development by using MTT and other methods. Finally, nude mouse xenograft models can be used to measure miRNA effects in vivo.

### Influence on cancer initiation

Tumorigenesis is attributed to a two-way interaction between cancer cells and the surrounding microenvironment rather than a tumor cell-autonomous mechanism triggered by accumulation of somatic aberrations [22].

Mesenchymal stem cells (MSCs), defined as multipotent stem cells that have the capacity to give rise to adipocytes, osteoblasts, and chondrocytes, are an important component of the tumor microenvironment [23]. Tumor cells can reprogram surrounding MSCs into tumor supportive myofibroblasts through intercellular communications, especially by releasing EVs. This long-time “education” by tumor cells contributes to the cancer initiation [24]. MiRNAs transferred by cancerous cell-derived EVs can promote cancer initiation. A positive feedback loop between multiple myeloma (MM) cells and MSCs is that MM cells promote the increase of miR146a in MSCs, leading to more cytokine secretion, which in turn favors MM cell growth and migration. MM cells secrete EVs containing miR-146a into MSC and the overexpression of miR-146a in MSC elevates secretion of several cytokines and chemokines including CXCL1, IL-6, IL-8, IP-10, MCP-1, and CCL-5, enhancing MM cell viability and migration as a result, which expands our knowledge of mutual communication between cells mediated by EV-associated miRNAs [25].

On the other hand, cancerous cells might discard anti-tumorigenic miRNAs via EVs to stimulate cancer initiation and progression [26]. For example, exosomal release of miR-202-3p from CLL cells into the microenvironment increases the expression of its target “suppressor of fused” (Sufu), a negative regulator of Hedgehog signaling, resulting in a decrease of its anti-tumorigenic effect [27]. Similarly, a metastatic gastric cancer cell line, AZ-P7a cells selectively secrete let-7 family miRNAs, which are considered mainly as tumor suppressor genes targeting oncogenes such as RAS and high-mobility group A2 (HMGA2), into the extracellular environment via exosomes to maintain their tumorigenic and metastatic propensities [21]. These results provide the basis for the hypothesis that cancerous cells specifically package tumor-suppressive miRNAs into exosomes to promote cancer initiation.

### Involvement in tumor angiogenesis

Angiogenesis refers to the formation of tumor-associated vessels, which is the result of an interplay between cancer cells and endothelial cells and results in the sprouting of locally pre-existing vessels or the recruitment of bone marrow-derived endothelial progenitor cells [26]. Recent studies have highlighted the functions of EV-associated miRNAs on angiogenesis and tumor development.

Vascular endothelial growth factor (VEGF) functions as an important factor in angiogenesis as they can bind to receptors to induce endothelial cell migration and form new blood vessels whose elevation significantly fosters angiogenesis and tumor development [28, 29]. MiR-21 in exosomes derived from transformed human bronchial epithelial (HBE) cells elevates levels of VEGF in HBE cells by activating STAT3, which promotes angiogenesis and malignant transformation of HBE cells [30].

Furthermore, miR-9 is a new star in regulating tumor angiogenesis by modulating the JAK-STAT pathway in endothelial cells. STAT proteins not only play a crucial role in tumor cell proliferation, survival, and invasion but also significantly contribute to the formation of a unique tumor microenvironment [31, 32]. Emerging evidence has shown that there exists a link between STATs activation in endothelial cells and tumor angiogenesis [33–35]. Exogenous miR-9 effectively reduces SOCS5 levels, leading to activated JAK-STAT pathway, which promotes endothelial cell migration and tumor neovascularization [36].

MiR-135b has also been found to be transferred into endothelial cells through exosomes by MM cells and target a factor-inhibiting hypoxia-inducible factor 1 (HIF1), thus enhancing angiogenesis [37].

### Promotion of tumor metastasis

Besides influencing located cells, cancer-derived EVs can affect cells in distant tissues and organs via delivering miRNAs.

Intravasation, the first step of metastasis cascade, allows the invasion of cancer cells through the basal membrane followed by local infiltration of the stroma-rich extracellular matrix (ECM), potentially leading to metastasis [26]. Modica and colleagues demonstrate an extracellular pro-tumorigenic role for tumor-derived, exosome-associated miR-939 that leads to an increase of monolayer permeability by targeting VE-cadherin and disrupting the endothelial barrier [38].

Additionally, exosome-mediated transfer of cancer-secreted miR-105 regulates tumor migration through targeting the tight junction protein ZO-1 and destroys these natural barriers against metastasis. This explains the phenomenon that overexpression of miR-105 in nonmetastatic cancer cells induces metastasis, whereas inhibition of miR-105 in highly metastatic tumors alleviates these effects [39].

Given that cell invasion is a key process in tumor metastasis, exosome-mediated miR-10b secretion is dramatically higher in metastatic breast cancer MDA-MB-231 cells than in nonmetastatic breast cancer cells or nonmalignant breast cells. MiR-10b suppresses the protein level of its target genes such as HOXD10 and KLF4, indicating their functional significance [40].

Brain metastasis leads to a particularly poor prognosis for cancer patients [41], with accumulating evidence suggesting that the destruction of the blood-brain barrier (BBB) is one of the key features of brain metastasis [42]. EVs can trigger the breakdown of BBB via delivery of miRNAs. For example, EVs containing miR-181c promote the destruction of BBB via degradation of its target gene, PDPK1, which leads to the downregulation of phosphorylated cofilin and the resultant-activated cofilin-induced modulation of actin dynamics [43].

Epithelial to mesenchymal transition (EMT) is reversible and the reciprocal mesenchymal to epithelial transition (MET) process allows cancerous cells to regain epithelial properties and integrate into distant organs, thus promoting long-distance metastasis [44, 45]. Members of the miR-200 family (miR-200a, miR-200b, miR-200c, miR-429, miR-141), which are enriched in the serum of patients with metastatic cancers, share the same seed sequence and the same targets. They have the ability of regulating MET process in large part by inhibiting the expression of Zeb1 and Zeb2, the transcriptional repressors of many epithelial genes [46]. Upon integrated into nonmetastatic cells, miR-200 microRNAs in EVs derived from metastatic cells alter gene expression and transfer metastatic capability [47].

Reprogrammed energy metabolism to fuel rapid cell growth and proliferation is an emerging hallmark of cancer [47]. It has been shown by Fong et.al that cancer cells are able to suppress glucose uptake by nontumor cells in the pre-metastatic niche, by secreting EVs abundant in miR-122. High miR-122 levels in the circulation are associated with metastatic progression in BC patients [48] and cancer cell-secreted miR-122 facilitates metastasis by increasing nutrient availability in the pre-metastatic niche [49]. MiR-122 suppresses glucose uptake by niche cells in vitro and in vivo by downregulating the glycolytic enzyme pyruvate kinase (PKM) [49].

Above all, EV-associated miRNAs can promote tumor long-distance metastasis through a wide range of mechanisms.

### Regulation of tumor immunity

The topic that miRNAs transferred by exosomes can regulate tumor immunity has now blossomed into a full-fledged field of research.

Dendritic cells (DCs) are typical antigen-presenting cells (APCs) which express a wide range of toll-like receptors (TLRs) and cytokines, playing an important role in activation of immune response [50]. TLRs lead to cell activation and cytokine production by recognizing and binding viral single-stranded RNA sequences on dendritic cells and B lymphocytes [51, 52].

Tumor-secreted miR-21 and miR-29a trigger a TLR-mediated prometastatic inflammatory response by binding as ligands to receptors of TLR family, namely murine TLR7 and human TLR8, in immune cells. This prompts the very first attempt at studying EV-associated miRNAs as paracrine agonists of TLRs and key regulators of the tumor microenvironment, suggesting their involvement in tumor-immune system communication and importance in tumor growth and spread [53].

Evidence has demonstrated that miR-203 may cause immune dysfunction as they can be transferred via exosomes to interfere with DCs and contribute to dysfunction of DCs by acting as the regulator of TLR4 and production of cytokines such as TNF-a and IL-12 [54]. In other circumstances, pancreatic cancer (PC)-derived exosomal miRNAs can inhibit mRNA expression of DCs and induce immune tolerance. Regulatory factor X-associated protein (RFXAP) is a key transcription factor for the MHC II gene whose deficiency can lead to a rare severe immunodeficiency disorder termed bare lymphocyte syndrome. MiR-212-3p transferred from PC-secreted exosomes downregulate RFXAP expression, inhibiting MHC class II expression and leading to inactivation of CD4+ T-lymphocytes [55].

### Contribution to drug resistance

Drug resistance represents a daunting challenge to the successful treatment of all kinds of cancers. Results from Yifang Wei are the first to show that secreted miR-221/222 acts as signaling molecules to mediate communication of tamoxifen resistance. MCF-7TamR exosomes enter into MCF-7wt cells where they release miR-221/222, and the elevated miR-221/222 effectively reduce the target gene expression of P27 and ERa, enhancing tamoxifen resistance in recipient cells [56].

Challagundla and colleagues have also identified that neuroblastoma cells secrete exosomic miR-21, leading to a TLR8 and NF-кB-dependent upregulation of miR-155. Exosomic miR-155 transferred by human monocytes was capable of directly targeting TERF1 and affecting telomerase activity and telomere length in NBL, which is involved in the increased chemoresistance CDDP. This novel exosomic miR-21/ TLR8/NF-кB/exosomic miR-155/TERF1 axis suggests that exosomes within the tumor microenvironment are important molecular targets to restore drug sensitivity [57].

While tumor-derived EVs modify the function of immune cells, immune cell-derived EVs can be used to treat cancer cells. An example is that DCs secrete EVs expressing functional major histocompatibility complex class I and class II, and *T* cell costimulatory molecules as antigen-presenting vesicles. Tumor peptide-pulsed DC-derived exosomes target specific cytotoxic T-lymphocytes in vivo and eradicate growth of established murine tumors in a T cell-dependent fashion, which can be utilized as cell-free vaccines for suppressing tumor growth [58].

### miRNAs transferred by noncancerous cell-derived EVs in cancer

We need to understand that tumors consist not only of malignant cells but also of a variety of stromal cell types including three subtypes: angiogenic vascular cells, infiltrating immune cells, and cancer-associated fibroblastic cells as well as ECM. MiRNAs in stromal cell-derived EVs make contributions to resisting cell death, avoiding immune destruction, activating invasion, inducing angiogenesis, and sustaining proliferative signaling [44, 59].

There exists a homeostatic cell-competitive system where normal epithelial cells secrete tumor-suppressive miRNAs via EVs to prevent the aberrant growth of neighboring cells. The failure of this system is considered to be a reason for tumor initiation [60–62]. Of these miRNAs, miR-143 has been shown to act as a death signal in the cell-competitive process and induce growth inhibition exclusively in prostate cancer cells in vitro and in vivo, which provides a novel insight into a tumor initiation mechanism [62].

MiR-21, a famous and well-studied microRNA, has been revealed to play a pivotal role in cancer proliferation, angiogenesis [30], tumor immunity [53], and drug resistance [57] as discussed above. Transferred from CAFs to the cancer cells, miR-21 suppresses ovarian cancer apoptosis and confers chemoresistance by binding to its direct novel target, APAF1. This leads to very open and constructive discussion that miR-21 delivered by exosomes derived from neighboring stromal cells in the omental tumor microenvironment can alter the malignant phenotype of metastatic ovarian cancer cells, indicating their potential function in tumor therapy [63].

### EV-associated lncRNAs in cancer

#### lncRNAs secreted by EVs

Long noncoding RNAs (lncRNAs) are RNA transcripts greater than 200 nucleotides in length, and they play important regulatory roles in gene expression [64, 65]. Once considered little more than genomic noise, recent observations have proved that lncRNAs can act as miRNA sponges [66] and mediate normal cellular processes through various mechanisms such as epigenetic regulation, chromatin remodeling, and transcriptional or post-transcriptional regulation or modulation of protein function and localization [67].

Several previously described lncRNAs such as MALAT1, HOTAIR, and GAS5 have been discovered to be expressed at higher levels within exosomes from HeLa and MCF-7 cells, suggesting that lncRNAs and exosomes may function together to disseminate cell signals that alter local cellular microenvironment and result in a phenotypic effect within the recipient cells (Table 2) [68].

**Table 2:** EV-associated lncRNAs in cancers

lncRNA	Cancer type	Biological function	Mechanism	Refs
lncRNA-p21	Prostate cancer	Suppress cancer initiation	Enhance drug resistance; suppress the genes regulated by p53	[71]
GAS5	Prostate cancer	Suppress cancer initiation	Suppress several anti-apoptotic genes	[72]
MEG3	Lung cancer	Suppress cancer initiation	Stimulate p53 expression	[73]
HOTAIR	Bladder cancer	Facilitate tumor progression	Regulate EMT and act as miR-205 sponge	[78]
MALAT1	Cervical cancer	Facilitate tumor progression	Modulate EMT	[79]
MONC	AKML	Facilitate tumor progression	Act as miR-99a/100~125b sponge	[81]
MIR100HG	AKML	Facilitate tumor progression	Same as lnc-MONC	[81]
H19	Liver cancer	Promote angiogenesis	Affect phenotype of endothelial cells	[86]
linc-ROR	HCC	Promote tumor progression	Inhibit p53 and act as miR-145 sponge	[88, 89]
linc-ROR	HCC	Enhance drug resistance	Elevate TGF level	[90]
Linc-VLDLR	HCC	Enhance drug resistance	Unknown	[67]
lncARSR	Renal cancer	Enhance drug resistance	Act as a ceRNA for miR-34 and miR-449	[91]

### Functions of EV-associated lncRNAs in cancer

LncRNAs can influence regulation of gene expression and have an impact on many different cancerous processes, where they can influence tumorigenesis, invasion, and metastasis.

The study of EV-associated lncRNAs begins with collecting tumor tissues and matched adjacent nontumor tissues. Then, lncRNA exposure levels are determined using RT-PCR and in situ hybridization and the exposure levels of the proteins are measured by western blot. In addition, dual-luciferase report assay can be performed to verify the target effect of lncRNAs on miRNAs. The proliferation, invasion, and migration ability of target cells after being infected were tested by MTT assay, wound healing assay, and transwell assays. Finally, researchers can utilize nude mouse xenograft models to measure lncRNA effects on tumor growth in vivo [69].

LncRNA-p21 and lncRNA-GAS5 are tumor suppressor molecules in the cellular machinery [70, 71]. Significantly higher level of exosomal lncRNA-p21 is observed in the patients with prostate cancer, which is stimulated by the p53 tumor suppressor protein. Upon transcription, it can suppress the expression of the genes transcriptionally regulated by p53 by binding to the hnRNP-K complex [71]. LncRNA-GAS5 secreted by prostate cancer cells is an important mediator of inducing apoptosis since it suppresses several anti-apoptotic genes by binding to the DNA-binding domain of the glucocorticoid receptor (GR), which prevents glucocorticoid response elements (GRE) from binding to the GR [72]. The lncRNA MEG3 is an example of another lncRNA that acts as a tumor suppressor gene. MEG3 functions by stimulating p53 expression and can also inhibit cell proliferation independent of the p53 protein [73].

EV-associated lncRNAs can foster tumor development through diverse mechanisms. Accumulating evidence has demonstrated that lncRNA-HOTAIR has the capability of facilitating tumor initiation and progression and is associated with poor prognosis in several cancers [74]. Importantly, lncRNA-HOTAIR functions by regulating several genes involved in EMT including snail family zinc finger 1 (SNAI1), laminin, beta 3 (LAMB3); laminin, gamma 2 (LAMC2); junctional adhesion molecule 2 (JAM2); and ABL proto-oncogene 2 (ABL2) [11, 75–77], as well as participating in the silencing of miR-205 expression in UBC cells through epigenetic regulation. MiR-205 targets the cell-cycle regulation gene cyclin J (CCNJ) and is proved relevant to the inhibition of proliferation, migration, and invasion of the urothelial bladder cancer cell lines [78].

LncRNA-MALAT1 is also involved in modulating EMT through regulating the expression of proteins concerning EMT, including E-cadherin, ZO-1, b-catenin, vimentin, and snail [79]. Another study indicates that lncRNA-MALAT1 promotes proliferation and invasion in cervical cancer cells by Hela and CaSki [80].

LincRNAs MONC and MIR100HG are mainly localized in the nucleus and highly expressed in acute megakaryoblastic leukemia (AMKL) blasts, whose expression is correlated with corresponding miR-99a/100~125b clusters, resulting in tumorigenesis and tumor development. It has been shown that MONC or MIR100HG knockdown inhibits leukemic growth of AMKL cell lines and cells from primary patient samples through a shRNA-induced loss-of-function study [81].

CD90 is a 25–37 kDa glycophosphatidylinositol-anchored protein involved in cell-to-cell and cell-matrix interaction, apoptosis, adhesion, migration, fibrosis, and cancer development [U]. Concerning the liver, CD90 is expressed by hepatic stem/progenitor cells [V] and is correlated with an aggressive phenotype during tumor growth, low differentiated HCC, and poor prognosis [82–85]. LncRNA H19, enriched in exosomes released by CD90+ cancer cells rather than parental hepatoma cells, plays an important role in the exosome-mediated phenotype of endothelial cells, thus promoting angiogenesis and cell-to-cell adhesion [86].

Linc-RoR is enriched in tumor cell-derived EVs during hypoxia and can modulate cellular signaling and cell survival in recipient cells as a stress-responsive lncRNA [87]. Linc-RoR prevents the activation of cellular stress pathways, such as the p53 response, promoting survival of iPSCs, and embryonic stem cells [88]. Linc-RoR has been shown to function as a miRNA sponge to miR-145 and modulate the expression of key effectors of the hypoxia response, such as HIF-1α expression, which contributes to the acute hypoxic response and can promote the expression of several hypoxia-inducible genes associated with angiogenesis, cell growth, differentiation, survival, and apoptosis [89]. Recent studies have identified a previously unrecognized role of linc-ROR as a mediator of cell-to-cell communication through the transfer of extracellular vesicles, resulting in acquired chemoresistance within tissues. EV-associated linc-ROR elevate transforming growth factor (TGF) level in recipient cells, which contributes to loss of therapeutic effect of agents such as sorafenib [90].

Another example of lncRNAs that enhance chemoresistance is linc-VLDLR. Takahashi and colleagues [67] have proposed the potential role of linc-VLDLR as a novel signaling mediator that can contribute to chemotherapeutic stress responses in hepatocellular cancer (HCC) through extracellular vesicle-mediated intercellular signaling and justified more studies to define the mechanisms, which are currently unknown. Besides, lncARSR has been found to be involved in receptor tyrosine kinase inhibitor sunitinib resistance in renal cancer [91]. Localized in the cytoplasm, lncARSR whose expression is correlated with clinical poor sunitinib response might function as a competing endogenous RNA (ceRNA) to sequester miRNAs, resulting in the liberation of corresponding miRNA-targeted transcripts [92, 93]. LncARSR could be packaged into exosomes and transferred to recipient cells to promote sunitinib resistance via competitively sponging miR-34/449 to promote AXL and c-MET expression in renal cancer cells [68].

### EV-associated circRNAs in cancer

CircRNAs are a class of novel endogenous ncRNAs that form a covalently closed continuous loop unlike linear ncRNAs [94] and provide new insights into the study of ncRNAs because of their tissue and developmental stage-specific expression [95]. Recently, circRNAs have been shown to function as miRNA sponges and RNA-binding protein (RBP) sequestering agents as well as transcriptional regulators to influence gene expression, which sets the foundation of the hypothesis that circRNAs transferred by EVs can take part in cell-to-cell communication with recipient cells [95, 96]. According to the research by Yan Li, abundant circRNAs are contained in EVs compared to the producer cells and changes of associated miRNA levels in producer cells may manipulate the sorting of circRNAs into EVs. For example, EV-associated circRNAs have been found to retain biological activity as growth suppressor by abrogating miR-7 [97]. The study of the mechanisms by which EV-associated circRNAs function in the process of cancer is still on the way. Hopefully, it will represent a research hotspot in the field of EV-associated ncRNAs.

### The future of EV-associated ncRNAs

As described above, the rapid development of EV-associated ncRNAs has contributed to reveal underlying mechanisms of cancer initiation and progression. However, there remain a large number of challenges.

The first issue is that since the current method is incapable of distinguishing between exosomes and microvesicles [6], it is controversial whether the function of EVs is related only to the ncRNAs that is encapsulated in them but not to its form. Therefore, more advanced technology should be used for separating and purifying each subtype of EVs.

Secondly, we should pay special attention to the tissue type being studied. The ncRNAs might serve as a tumor suppressor in one cell type and an oncogene in another. For example, in general, let-7 miRNAs act as a tumor suppressor by targeting oncogenes such as RAS and HMGA2 and let-7 miRNAs are downregulated in many cancers from solid organs [98], while they act as oncogenes since a metastatic gastric cancer cell line named AZ-P7a releases let-7 miRNAs into the extracellular environment to maintain their oncogenesis and invasiveness [21]. Therefore, it is important and necessary to clarify what tissue type AZ-P7a cells represent in the research.

The third issue is lacking of a universal method to analyze EV-associated ncRNAs which often leads to discrepancies between studies performed by different groups. Besides, occurrence of EMV-mediated ncRNA transfer has been indirectly confirmed by detecting the altered expression levels of internal miRNAs in both donor and recipient cells. In the future, fluorescence signal amplification by a confocal imaging system may open new avenues for studying EMV transfer directly [99].

EVs are secreted by most cell types and exist in various body fluids including the blood, urine, and saliva. NcRNAs contained in EVs represented the biological or pathological states of cells. Because of their resistance to endogenous RNase and high stability under different storage conditions, EV-associated ncRNAs can serve as valuable noninvasive biomarkers for the diagnosis and prognosis of cancers including glioblastoma melanoma, liver cancer, gastric cancer, ovarian cancer, breast cancer, lung carcinoma, and so on (Table 3) [100–107]. Importantly, panels consisting of a collection of several ncRNAs rather than single miRNA, lncRNA, or circRNA will be necessary to precisely evaluate the diagnosis and prognosis of any kind of cancer [74].

**Table 3:** Current available EV-associated ncRNAs as tumor biomarkers

EV-associated ncRNA	Cancer type	Biological function	Refs
miR-223	Breast cancer	Promote invasion	[100]
let-7 family	Gastric cancer	Suppress cancer initiation	[21]
miR-17-92	Leukemia	Enhance migration and tube formation	[101]
miR-15	MM	Facilitate progression	[102]
miR-125b	Melanoma	Monitor indicator	[103]
miR-16	HCC	Mediate intercellular communication	[104]
miR-21	Breast cancer	Monitor indicator	[108]
miR-21/155	Neuroblastoma	Enhance drug resistance	[57]
H19	Liver cancer	Promote angiogenesis	[86]
HOTAIR	Bladder cancer	Facilitate tumor progression	[78]
MALLATl	Cervical cancer	Facilitate tumor progression	[79]
MEG3	Lung cancer	Suppress cancer initiation	[73]
GAS5	Prostate cancer	Suppress cancer initiation	[72]
lincRNA-ROR	HCC	Promote tumor progression	[88, 89]
lnc-ATB	HCC	Promote metastasis	[106]
lnc-PVT1	HCC	Promote cell proliferation	[107]

EV-associated ncRNAs have also provided new opportunities for the treatment of cancer. EVs are in small size and capable of crossing biological membranes and protect their cargo from degradation, which suggests that they are ideal delivery systems for the transfer of specific molecules such as miRNAs or anti-miRNAs [108].

For example, it has been discovered that miR-122-transfected adipose tissue-derived MSC (AMSC) can effectively package miR-122 into secreted exosomes rendering cancer cells sensitive to chemotherapeutic agents through alteration of miR-122-target gene expression in HCC cells by mediating miR-122 communication between AMSCs and HCC cells. Furthermore, intra-tumor injection of 122-Exo significantly increased the antitumor efficacy of sorafenib on HCC in vivo which suggests that the export of miR-122 via AMSC exosomes represents a novel strategy to enhance HCC chemosensitivity and can be used for HCC therapy [109].

Recently, microvesicles have also been used as the shuttle of delivering antisense RNA targeted to miR-150 that is proved as an oncomir by regulating the VEGF secretion of TAMs into mice to treat tumors, leading to a new avenue for the transfer of antineoplastic drugs [110].

However, the therapeutic potential of EV-associated ncRNAs in cancer is largely unknown. The intracellular delivery of EV-associated proteins can be fulfilled by a new tool named “exosomes for protein loading via optically reversible protein–protein interactions” (EXPLORs) [111]. Similar tools may be also utilized in the study of EV-associated ncRNAs and further research is in urgent need before used in clinic.

## Conclusions

EVs, previously largely thought to function like garbage bags to remove excess or unnecessary constituents from the cells, have now been discovered to be mediators in specific cell-to-cell communication via transferring proteins, RNAs and DNAs. Exosomes are the most widely studied EVs. They can transfer information to the target cells through three main ways: receptor-ligand interaction, direct fusion with plasma membrane, and endocytosis by phagocytosis [112]. Amongst the components they transfer, ncRNAs have drawn the great interest of researchers since emerging evidence has suggested that EV-associated ncRNAs fulfill important functions in the regulation of gene expression and participate in the process of versatile diseases, particularly in cancer. NcRNAs derived from both cancerous cells and noncancerous cells influence on tumorigenesis, angiogenesis, metastasis, immunity, and drug resistance through diverse mechanisms. However, an outstanding question remains as to whether EV-associated ncRNAs actually function in vivo and more research utilizing convenient in vivo model systems are needed as a result. Further studies will likely also focus on the potential role of EV-associated ncRNAs as hopeful diagnostic biomarkers and novel treatment modalities, which will contribute to the health of human beings.

## Abbreviations

ABL2: ABL proto-oncogene 2
APCs: Antigen-presenting cells
BBB: Blood-brain barrier
CCNJ: Cell-cycle regulation gene cyclin J
circRNAs: Circular RNAs
CLL: Chronic lymphocytic leukemia
DCs: Dendritic cells
ECM: Extracellular matrix
EMT: Epithelial to mesenchymal transition
EVs: Extracellular vesicles
FIH: Factor-inhibiting hypoxia-inducible
GAS5: Growth arrest-specific 5
GR: Glucocorticoid receptor
GRE: Glucocorticoid response elements
HBE: Human bronchial epithelial
HCC: Hepatocellular cancer
HMGA2: High-mobility group A2
HOTAIR: HOX antisense intergenic RNA
JAM2: Junctional adhesion molecule 2
LAMB3: Laminin, beta 3
LAMC2: Laminin, gamma 2
Linc-RoR: Long intergenic nonprotein coding RNA, regulator of reprogramming
linc-VLDLR: The large intergenic noncoding RNA-VLDLR
lncARSR: lncRNA activated in RCC with sunitinib resistance
lncRNAs: Long noncoding RNAs
MALAT1: Metastasis-associated lung adenocarcinoma transcript 1
MET: Mesenchymal to epithelial transition
miRNAs: MicroRNAs
MM: Multiple myeloma
MSC: Mesenchymal stromal cells
ncRNAs: Noncoding RNAs
PC: Pancreatic cancer
piRNAs: Piwi-interacting RNAs
RBP: RNA-binding protein
RFXAP: Regulatory factor X-associated protein
SNAI1: Snail family zinc finger 1
Sufu: Suppressor of fused
TGF: Transforming growth factor
TLRs: Toll-like receptors
VEGF: Vascular endothelial growth factor.
